# New Approaches for Early Detection and Assessment of Alzheimer's Disease: An Analysis of Recent Studies

**DOI:** 10.1002/alz70856_102396

**Published:** 2025-12-25

**Authors:** Juliana de Oliveira Alves

**Affiliations:** ^1^ Universidade Feevale, Novo Hamburgo, Rio Grande do Sul, Brazil

## Abstract

**Background:**

Alzheimer's disease (AD) and other dementias pose a significant global challenge, with a projected substantial increase in prevalence over the coming decades (JANNATI, 2024). Early detection of AD, particularly during the preclinical stage, is crucial for implementing effective interventions to slow disease progression (ALTY, 2024). However, traditional cognitive assessment methods, such as the Mini‐Mental State Examination (MMSE), have notable limitations, including low sensitivity to mild cognitive changes and susceptibility to demographic factors like age and education level (ALTY, 2022). Furthermore, these tests are often time‐consuming and require specialized training for administration (JANNATI, 2024). These challenges underscore the urgent need for more sensitive, rapid, and accessible tools for dementia screening, particularly in primary care settings (ALTY, 2024).

**Method:**

This analysis reviewed three studies published within the past five years, retrieved from the Medline database, focusing on novel approaches for early detection of AD. The first study evaluated the efficacy of the Digital Clock and Recall (DCR) test compared to the MMSE in identifying mild cognitive impairment (MCI) and early dementia. The second study investigated TapTalk, a smartphone‐based test that combines hand movement and speech analysis with blood biomarkers such as *p*‐tau181 to assess preclinical AD risk. The third study validated the TAS Test, an online tool that integrates motor‐cognitive functions and correlates them with *p*‐tau181 levels to classify preclinical AD risk.

**Result:**

The studies revealed promising results. The DCR demonstrated higher accuracy and sensitivity than the MMSE in detecting MCI, requiring less than three minutes to administer and being less influenced by demographic factors. TapTalk identified hand movement and speech patterns associated with preclinical AD risk, showing potential as a non‐invasive and accessible tool. The TAS Test effectively detected subtle motor changes that may precede cognitive symptoms, highlighting its usefulness in early AD stages.

**Conclusion:**

These studies emphasize the potential of digital assessments and biomarkers as innovative, sensitive, and accessible methods for early detection of AD. Their large‐scale application could transform dementia screening and enable earlier, more effective interventions, particularly in primary care settings.

